# *KCNJ14* knockdown significantly inhibited the proliferation and migration of colorectal cells

**DOI:** 10.1186/s12920-022-01351-4

**Published:** 2022-09-13

**Authors:** Bin Li, Ning Ge, Zhongping Pan, Chaofeng Hou, Kun Xie, Dongfang Wang, Junwei Liu, Jie Wan, Feihong Deng, Mengyi Li, Shuping Luo

**Affiliations:** Department of Colorectal Surgery, Zhengzhou Central Hospital Affiliated To Zhengzhou University, 16 Tongbai North Road, Zhengzhou, Henan China

**Keywords:** Colorectal cancer, *KCNJ14*, Prognosis, Methylation, Biological target

## Abstract

**Background:**

This study attempted to verify the potential of *KCNJ14* as a biomarker in colorectal cancer (CRC).

**Methods:**

Data on transcriptomics and DNA methylation and the clinical information of CRC patients were downloaded from The Cancer Genome Atlas and Gene Expression Omnibus databases. Biological information analysis methods were conducted to determine the role of *KCNJ14* in the prognosis, diagnosis, immune cell infiltration, and regulation mechanism of CRC patients. The effect of *KCNJ14* on the proliferation and migration of HCT116 and SW480 CRC cell lines was verified by in vitro experiments (MTT, colony-forming, wound healing, and transwell assays). Western blotting was performed to detect the effect of *KCNJ14* on the levels of mTOR signalling pathway-related proteins.

**Results:**

*KCNJ14* expression was remarkably increased in CRC tissues and cell lines, which reduced the overall survival time of patients. *KCNJ14* mRNA was negatively regulated by its methylation site cg17660703, which can also endanger the prognosis of patients with CRC. Functional enrichment analysis suggested that *KCNJ14* is involved in the mTOR, NOD-like receptor, and VEGF signalling pathways. *KCNJ14* expression was positively correlated with the number of CD4 + T cells and negatively correlated with that of CD8 + T cells in the immune microenvironment. *KCNJ14* knockdown significantly reduced not only the proliferation and migration of CRC cell lines but also the levels of mTOR signalling pathway-related proteins.

**Conclusions:**

This study not only increases the molecular understanding of *KCNJ14* but also provides a potentially valuable biological target for the treatment of colorectal cancer.

**Supplementary Information:**

The online version contains supplementary material available at 10.1186/s12920-022-01351-4.

## Background

Colorectal cancer is the fourth most lethal cancer worldwide, and its incidence is expected to increase to 2,500,000 cases in 2035 [1]. Evidence suggests that the development of CRC is due to hereditary and environmentally harmful factors and long-standing inflammatory bowel disease [2]. Owing to the complex pathogenesis of colorectal cancer, it is difficult to improve the prognosis of patients despite the comprehensive treatment by surgery and adjuvant radiotherapy [3]. Therefore, a treatment scheme that improves the prognosis of patients with colorectal cancer is urgently required. Previous studies have suggested that biologically targeted therapy may be ideal for the treatment of malignant tumours [4]. However, the basic premise of biologically targeted therapy is to identify key regulatory genes that affect the prognosis of patients with malignant tumours.

*KCNJ14*, also known as IRK4 and KIR2.4, has been mapped to the chromosomal locus 19q13, and the encoded protein has been identified to belong to a family of integral membrane proteins that act as ATP-sensitive inward rectifier potassium (K^+^) channels [5]. Central to the entire discipline of inward rectifier K^+^ channels is the concept of regulation of K^+^ flow into cells at potentials negative to the potassium equilibrium potential [6]. Hence, this class of proteins is a major interest of researches focusing on multiple biological processes, such as heart rate regulation, neurotransmitter release, epithelial electrolyte transport, and participation in immune regulation [7]. Recent advances in inward rectifier K+ channels have heightened the need for cancer research on lung cancer [8], neuroblastoma [9], and glioblastoma [10]. However, only few studies have explored on the regulatory effect of *KCNJ14* on the pathological process of malignant tumours, especially in the prognosis of patients with colorectal cancer.

Therefore, this study attempted to investigate the effect of *KCNJ14* on the prognosis of patients with colorectal cancer and evaluate its regulatory relationship with the complex pathological process of colorectal cancer. First, transcriptomic expression data, DNA methylation data, and the detailed clinical characteristics of patients with colorectal cancer were collected from the public database to explore the changes in *KCNJ14* expression in colorectal cancer and the relationship between *KCNJ14* expression and the clinical characteristics of patients. Subsequently, we verified that *KCNJ14* knockdown could significantly reduce the proliferation and migration of colorectal cancer cell lines and revealed the regulatory mechanism of *KCNJ14* leading to poor prognosis in colorectal cancer. To our knowledge, this is the first study to investigate the potential mechanism of *KCNJ14* in colorectal cancer. From a genetic perspective, we identified a novel biological target for diagnosis, treatment, and prognosis of patients with CRC.

## Methods

### Data collection

TCGA transcriptome profiling data and the clinical information of 488 patients with colorectal adenocarcinomas and 42 adjacent tissues (workflow type: HTSeq-FPKM) were collected from the Genomic Data Commons (GDC) Data Portal (https://portal.gdc.cancer.gov/). Those with missing clinical data, such as age, sex, lymphatic invasion, pathological TNM classification, tumour stage, overall survival (OS), and survival status, were excluded. Thus, 339 patients with complete clinical information were included in the analysis (Table 1). DNA methylation data of colorectal cancer patients were downloaded to further explore the effects of *KCNJ14* expression. We also downloaded microarray datasets for GSE50117 based on the GPL6480 platforms from the GEO database (http://www.ncbi.nlm.nih.gov/geo), which contained nine paired tumour-normal colorectal samples, to explore the changes in *KCNJ14* expression in colorectal cancer [11]. GSE31595, based on the GPL570 platform, had 37 tissue samples of colorectal cancer and the survival status of patients [12]. The GSE31595 and TCGA transcriptomic data were used to perform a meta-analysis.

**Table 1:** The detailed clinical features of CRC patients in TCGA database

Covariates	Type	Total	Percentages (%)
Age	<=65	146	43.07
	>65	193	56.93
Gender	Female	159	46.9
	Male	180	53.1
Lymphatic invasion	No	203	59.88
	Yes	136	40.12
Pathologic_M	M0	269	79.35
	M1	44	12.98
	M2	26	7.67
Pathologic_N	N0	197	58.11
	N1	84	24.78
	N2	58	17.11
Pathologic_T	T1	9	2.65
	T2	60	17.7
	T3	232	68.44
	T4	38	11.21
Tumor_stage	Stage I	59	17.4
	Stage II	135	39.82
	Stage III	100	29.5
	Stage IV	45	13.28
KCNJ14 expression	High	170	50.15
	Low	169	49.85

### Meta-analysis

To date, no study has explored the prognostic value of *KCNJ14* in colorectal cancer; thus, herein, we performed a meta-analysis to evaluate the importance of *KCNJ14* expression in the prognosis of patients from both databases. OS was considered a prognostic outcome, and the prognostic significance of *KCNJ14* expression is shown as hazard ratios (HRs) with 95% confidence interval (CIs). The *Q* test (*I*^2^ statistics) was used to evaluate heterogeneity between the two databases. A fixed effects model was chosen because there was no statistical heterogeneity (*I*^2^ < 50%, *P* ≥ 0.1). Otherwise, when there was statistical heterogeneity between the two databases (*I*^2^ > 50%, *P* < 0.1), a random-effects model was used [13].

### TIMER database analysis

The Tumour Immune Estimation Resource (TIMER; https://cistrome.shinyapps.io/timer) is an interactive database that provides comprehensive computation and visualisation of the inextricable relationship between tumour immunologic and genetic information, such as gene expression, mutation, and copy number variants [10]. In this study, we evaluated the association between *KCNJ14* expression and the infiltration of six different immune cell types (B cells, CD4 + T cells, CD8 + T cells, macrophages, neutrophils, and dendritic cells).

### GO and KEGG analysis

Gene ontology (GO) enrichment and Kyoto Encyclopedia of Genes and Genomes (KEGG) analyses were performed to explore the biological processes and signalling pathways associated with *KCNJ14* expression in colorectal cancer. First, we divided the patients into two groups based on median *KCNJ14* expression as follows: high and low *KCNJ14* expression groups. We then selected the differentially expressed genes between the two groups, and R software (v.4.0.3) was used to analyse the significant biological functions and critical pathways related to *KCNJ14*. Results were considered significantly enriched when *P* < 0.05.

### CMap analysis

The connectivity map (CMap, https://portals.broadinstitute.org/CMap/) is a public database that contains drug-induced gene expression profiles and reveals the biological connection between genes, drugs, and diseases [14]. We used CMap analysis to select candidate drugs for colorectal cancer by comparing the transcriptome data with database information. Genes with positive and negative relationship with *KCNJ14* were selected to obtain information on alternative drugs by accessing the CMap database. Four candidate drugs were selected according to the most negative enrichment index, while setting *P* < 0.001 as the filter condition. The corresponding chemical structure formulas were obtained from the publicly available PubChem database (https://pubchem.ncbi.nlm.nih.gov/).

### Cell treatment and RT-PCR

The human normal colorectal mucosal cell line FHC and the colorectal cancer cell lines HCT116 and SW480 were purchased from the American Type Culture Collection (ATCC) and cultured in RPMI 1640 medium with 10% foetal bovine serum (FBS) in a 5% CO_2_ cell incubator at a constant temperature of 37 °C. Both HCT116 and SW480 cell lines were treated with S-adenosyl methionine at concentrations of 100 μM and 200 μM or decitabine at 5 μM and 10 μM, respectively, and expression of *KCNJ14* was verified by RT-PCR. Total RNA was extracted from cells using TRIzol reagent (Invitrogen, US) and reverse-transcribed into cDNA. We performed RT-PCR to evaluate *KCNJ14* expression using specific primers as follows: 5′-GGGGTCCCTCCGTCCAAT-3′ (sense) and 5′-CAGTGCCCGTCTTTCTTGAC-3′ (antisense)*.* In addition, HCT116 and SW480 cells were transfected with the negative control shRNA (NC) or *KCNJ14-*targeted shRNA (shKCNJ14: 5′-GATCCGCCAGGATGTGGATGTGGGCTTTGATTTCAAGAGAATCAAAGCCCACATCCACATCCTGGTTTTTTGGAAG-3′) for 24 h and used for subsequent experiments.

### Western blotting

The shRNA-treated HCT116 and SW480 cells were collected and lysed in RIPA buffer containing protease and phosphatase inhibitors. After incubation on ice for 30 min, cell lysates were centrifuged at 12,000 rpm at 4 ℃ for 15 min. An amount of 25 μL 4 × protein loading buffer was added to 75 μL supernatant after quantification of protein concentration. The mixed protein samples were analysed by SDS-PAGE and transferred onto polyvinylidene fluoride (PVDF) membranes. Next, the membrane was sealed with skimmed milk at room temperature for 1 h and incubated with specific primary antibodies against mTOR signalling pathway-related proteins (*KCNJ14*, Cat 14-171-1-AP, Proteintech; phospho-AKT, Cat 66444-1-Ig, Proteintech; AKT, Cat 60203-2-Ig, Proteintech; Phospho-mTOR, Cat 67778-1-Ig, Proteintech; and mTOR, Cat 66888-1-Ig, Proteintech) at 4 °C overnight. After washing with TBST, the membranes were incubated with the corresponding secondary antibodies conjugated with horseradish peroxidase (HRP) at 25 °C in the dark for 1 h. Finally, band signals were detected using a chemiluminescence (ECL) detection system.

### MTT and colony-forming experiment

The shRNA-treated HCT116 and SW480 cells were inoculated in 96 well plates at a density of 2000 cells per well, 20 μL MTT was added to each well, and cells were incubated at 37 ℃ for 4 h. After which, 150 μL DMSO was added to the wells, and further incubation for 15 min in the dark was performed to dissolve the formed formazan crystals. Finally, the absorbance of the treated SW480 cells was measured using a microplate reader at 490 nm. The number of HCT116 and SW480 cells transfected with shRNA was calculated to be 10^3^ for culture in 60 mm dishes with complete medium. After 14 days, the cells were washed with PBS, fixed with paraformaldehyde, and stained with a crystal violet solution. Finally, the number of cell colonies was recorded and calculated.

### Wound healing assay and Transwell assay

HCT116 and SW480 cells were cultured in six-well plates and treated with either NC or shKCNJ14. When the cells reached 95% confluence, a sterile pipette tip was used to wound them. After washing with PBS, the cells were continuously cultured for 48 h in serum-free medium. The relative distances between cells at the same location were recorded by photography. HCT116 and SW480 cells were treated with shRNA and resuspended in a medium containing 5% serum. A total of 10^5^ cells were selected and cultured in the Transwell chamber, and 500 μL of medium with 20% serum was added to the lower chamber of the 24-well plate. Cells that invaded the bottom surface of the transwell chamber after 48 h were stained with crystal violet and photographed after fixation.

### Statistical analysis

R software (v.4.0.3) was used to analyse the raw data from the TCGA and GEO databases. The differential expression of *KCNJ14* in colorectal and adjacent tissues was analysed using a *t*-test. Therein, 42 normal mucosae and their corresponding tumour tissues were further assessed using matched-pair analysis. Pearson’s correlation coefficient was used to identify co-expressed genes and the relationship between *KCNJ14* expression and DNA methylation. Moreover, we used Kaplan–Meier curves to evaluate the association between patient survival and *KCNJ14* expression, as well as *KCNJ14* DNA methylation. Receiver operating characteristic (ROC) analysis was used to assess the diagnostic significance of *KCNJ14*. In addition, we utilised univariate and multivariate Cox regression models to estimate whether *KCNJ14* is an independent prognostic factor for colorectal cancer. Finally, the results obtained from in vitro experiments are presented as mean ± SD, and a *t*-test and a one-way analysis of variance (ANOVA) test were used for statistical analysis. *P*-value less than 0.05 was considered statistically significant.

## Results

### KCNJ14 mRNA expression is abnormally increased in colorectal cancer

**Fig. 1:**
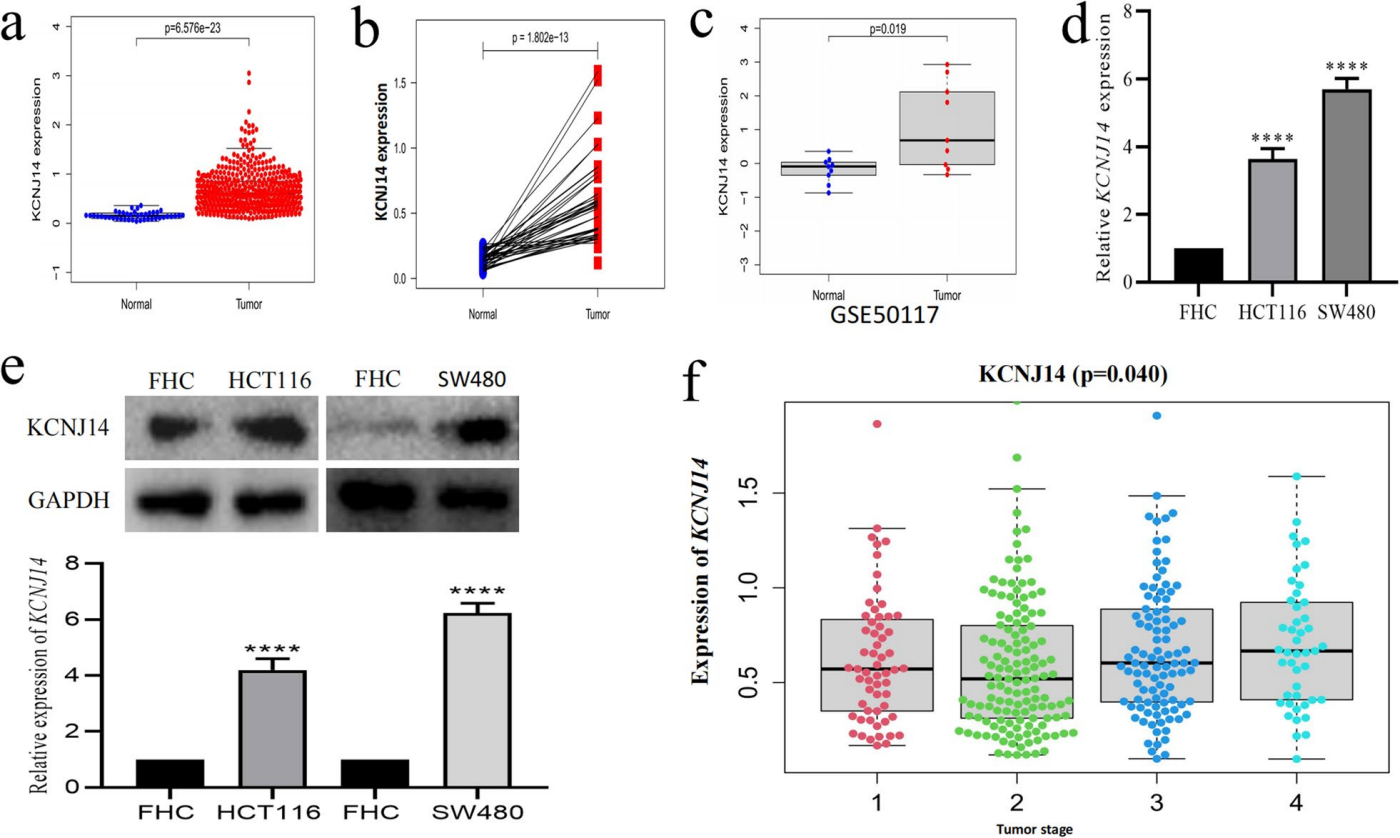
*KCNJ14* mRNA and protein levels in colorectal cancer cells and tissues and normal cells and adjacent tissues. **a** The mRNA level of *KCNJ14* in colorectal cancer and adjacent tissue samples based on the TCGA database. **b** The mRNA level of *KCNJ14* in 42 pairs of tumor-normal samples based on the matched-pair analysis. **c** The mRNA level of *KCNJ14* in nine paired tumor-normal colorectal samples of GSE50117. **d** The mRNA level of *KCNJ14* in normal human colonic epithelial cells (FHC) and colorectal cancer cell lines (HCT116 and SW480). **e** The protein level of *KCNJ14* in three cell lines. **f** The correlation between *KCNJ14* expression and tumour stage in CRC. ****P < 0.0001, compared with the FHC group

To explore the difference in *KCNJ14* expression in colorectal cancer tissues [15], we obtained colorectal cancer and adjacent tissue samples from the TCGA (cancer: 488 cases; adjacent: 42 cases) and GSE50117 (cancer: 9 cases; adjacent: 9 cases) databases, respectively. The results showed that the mRNA expression level of *KCNJ14* increased significantly in both the TCGA (*P* < 0.001) and GSE50117 (*P* = 0.019) datasets (Fig. 1a, c). In addition, we conducted a matched-pair analysis to process 42 pairs of tumour-normal samples and obtained a similar result (*P* < 0.001) (Fig. 1b).

To verify the reliability of the results, we used RT-qPCR to evaluate the mRNA expression of *KCNJ14*. We found that HCT116 and SW480 CRC cells had a significantly upregulated *KCNJ14* mRNA expression compared to the normal cell line (Fig. 1d). Western blotting revealed that the protein levels of *KCNJ14* in HCT116 and SW480 CRC cells are significantly increased compared to those in the normal cell line (Fig. 1e). More importantly, the expression of *KCNJ14* increased with an increase in tumour stage (*P* = 0.040) (Fig. 1f), and the increase in tumour stage of patients with colorectal cancer was a poor prognostic factor [16]. Collectively, these results suggest that *KCNJ14* may have an important regulatory effect on the pathological process of colorectal cancer.

### High KCNJ14 mRNA level is regulated by its DNA methylation in colorectal cancer

To determine why the expression of *KCNJ14* is abnormally increased in colorectal cancer cells, we downloaded the DNA methylation data of patients with colorectal disease from the TCGA database. Based on the important regulatory effect of DNA methylation on the expression of downstream mRNA [17], from a large number of DNA methylation sites, we selected 11 CpG sites with regulatory effects on *KCNJ14* expression (Additional file 1: Fig. S1a). Subsequently, we conducted a co-expression analysis and found that the methylation level of cg17660703 is positively correlated with *KCNJ14* (Additional file 1: Fig. S1b). However, the methylation of the remaining 10 CpG sites showed no significant association. The high methylation status of cg17660703 was negatively correlated with overall survival (*P* = 0.006) (Additional file 1: Fig. S1c). Based on these results, the high expression of *KCNJ14* in colorectal cancer may be positively regulated by the methylation site of cg17660703.

### High expression of KCNJ14 can independently affect poor prognosis of patients

**Fig. 2:**
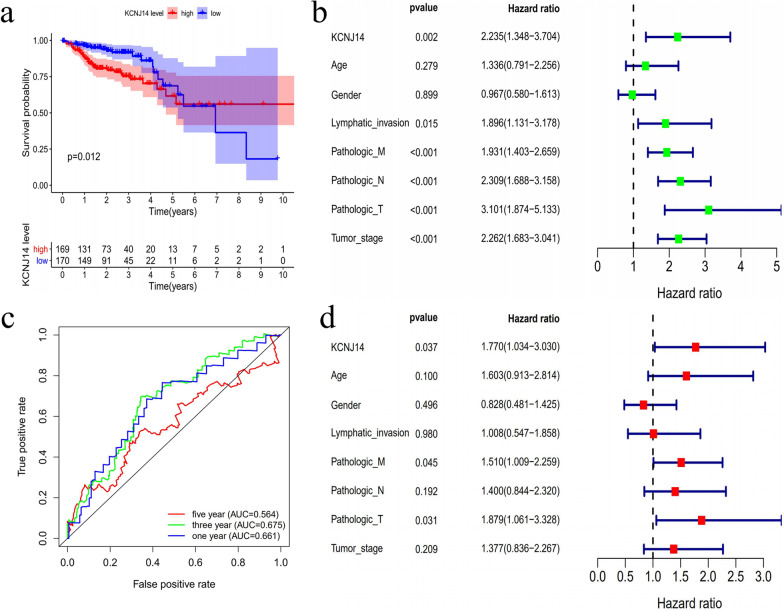
Association between *KCNJ14* expression and prognosis of CRC patients. **a** The overall survival of CRC patients with high or low expression of *KCNJ14.* **b** Results of univariate analysis of clinical characteristics related to CRC. **c** The relationship between of *KCNJ14* expression and 1-year, 3-year, and 5-year survival rates of CRC patients. **d** Results of multivariate analysis of clinical characteristics related to CRC

Based on the abnormally high expression of *KCNJ14* in colorectal cancer, we further explored its influence on patient prognosis. First, we used Kaplan–Meier curves to assess the correlation between high expression of *KCNJ14* and patient survival and found that high *KCNJ14* expression is associated with shorter overall survival of patients (*P* = 0.012) (Fig. 2a). Subsequently, further subtype analysis showed that the high expression of *KCNJ14* could significantly reduce the disease-free survival (stage I to III) (Additional file 2: Fig. S2a) and progression-free survival (stage IV) of patients (Additional file 2: Fig. S2b).

ROC and AUC were used to estimate the diagnostic value of *KCNJ14* (Fig. 2c). To further improve the reliability of our results, we used Cox regression models to predict the effect of *KCNJ14* on the prognosis of patients. Univariate analysis showed that *KCNJ14* and several clinical characteristics, such as lymphatic invasion, pathological TNM stage, and tumour stage (hazard ratio [HR] > 1; *P* < 0.05), are significantly related to OS (Fig. 2b). Multivariate analysis showed that *KCNJ14,* pathological T stage, and pathological M stage can independently affect the prognosis of colorectal patients (HR > 1; *P* < 0.05; Fig. 2d). Collectively, these results suggest that high *KCNJ14* expression is an independent risk factor for the prognosis of patients and may play a pathogenic role in colorectal cancer.

### KCNJ14 knockdown significantly inhibits the biological behaviour of colorectal cancer cell lines

**Fig. 3:**
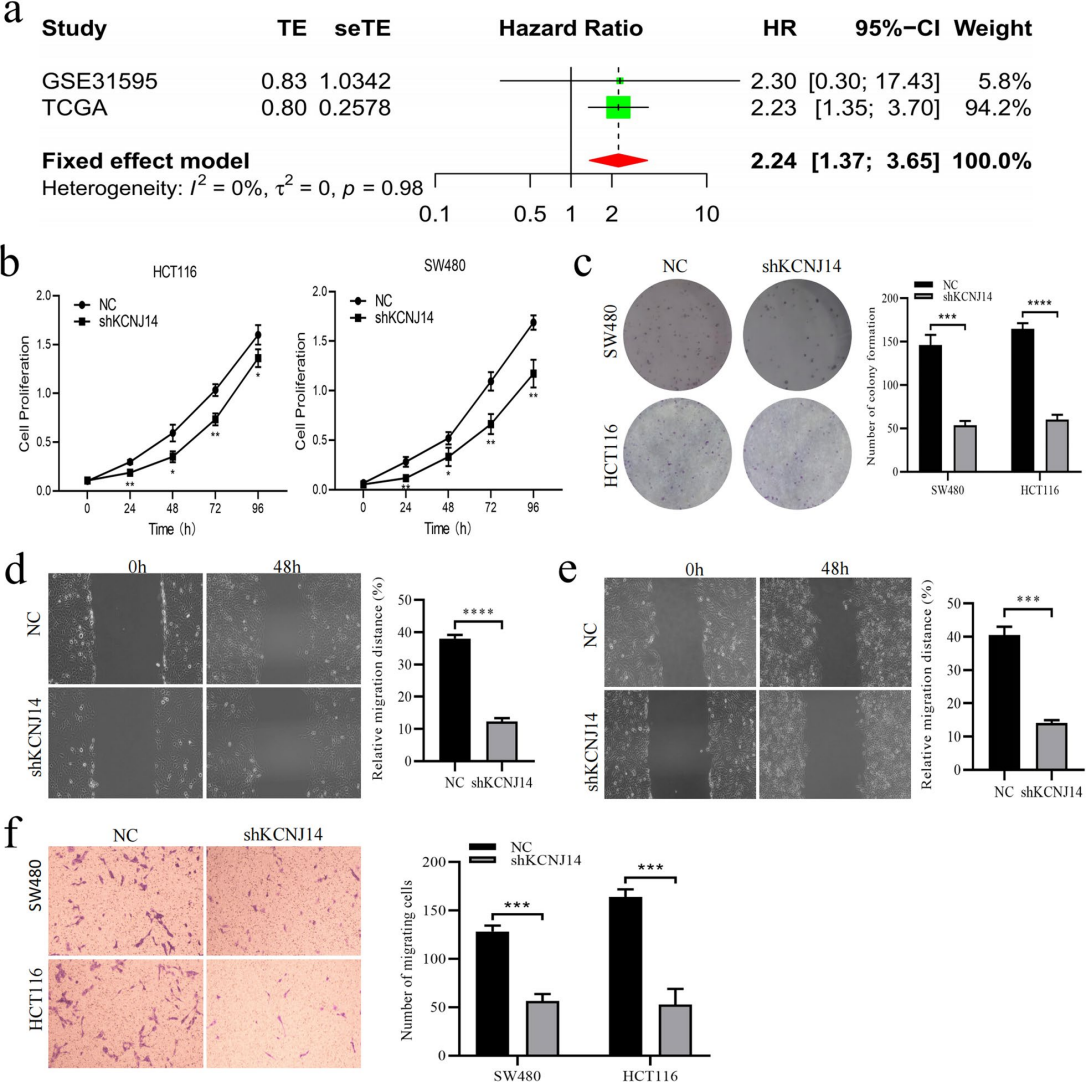
*KCNJ14* knockdown remarkably reduced the proliferative and invasive capacities of CRC cells. **a** Forest plot of high *KCNJ14* expression with unfavourable OS in CRC patients from two databases based on a meta-analysis. **b** Proliferation curves of HCT116 and SW480 cells treated with NC or shKCNJ14. **c** Staining results of cell colonies in NC or shKCNJ14 group of HCT116 and SW480 cells. **d**, **e** The relative distances of the wound healing assay of HCT116 and SW480 cells treated with NC or shKCNJ14. **f** Staining results of the Transwell assay in NC or shKCNJ14 group of HCT116 and SW480 cells. ***P < 0.001, ****P < 0.0001 vs NC group

We further verified the adverse effects of *KCNJ14* expression on the prognosis of patients with colorectal cancer. A meta-analysis found no significant heterogeneity between the two databases (*I*^2^ = 0%, *P* = 0.98); thus, a fixed-effect model was applied. Because the pooled HR for the correlation between high *KCNJ14* expression and patient OS was 2.24 (95% CI: 1.37–3.65), we can conclude that *KCNJ14* high expression is an independent predictor of unfavourable OS in patients with colorectal cancer (Fig. 3a).

To further verify the effect of *KCNJ14* on the biological behaviour of colorectal cancer cells, we used gene interference technology to reduce the protein and mRNA levels of *KCNJ14* in two colorectal cancer cell lines (Additional file 3: Fig. S3). We found that knocking down *KCNJ14* significantly inhibits the proliferation of HCT116 and SW480 cells (Fig. 3b, c). In addition, wound healing assay results showed that knockdown of *KCNJ14* significantly inhibits the migration of HCT116 (Fig. 3d) and SW480 (Fig. 3e) cell lines. Finally, Transwell assay results suggested that *KCNJ14* knockdown can reduce the invasive ability of cancer cells (Fig. 3f). Collectively, these results suggest that knockdown of *KCNJ14* expression can significantly inhibit the malignant behaviour of cancer cells in the pathological process of colorectal cancer.

### Regulatory mechanism of KCNJ14 in the pathological process of colorectal cancer

**Fig. 4:**
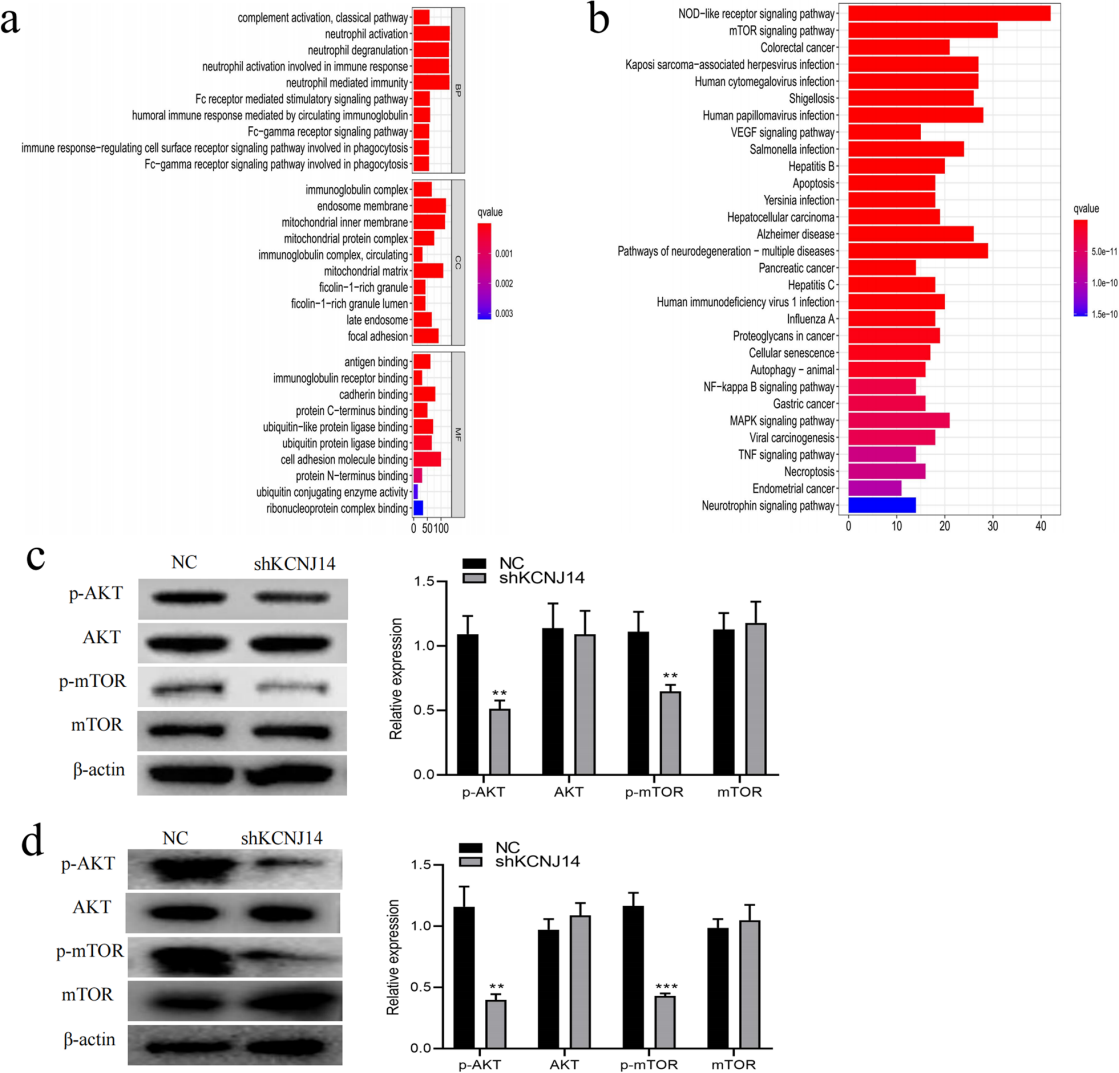
*KCNJ14* knockdown significantly inhibited the mTOR signalling pathway. **a** GO annotation of *KCNJ14*. BP: biological process, CC: cell component, MF: molecular function. **b** KEGG analysis of the biological functions of *KCNJ14.* **c** The levels of mTOR signalling pathway-related proteins in NC and shKCNJ14 groups of SW480 cells. **d** The levels of mTOR signalling pathway-related proteins in NC and shKCNJ14 groups of HCT116 cells. **P < 0.01, ***P < 0.001 vs NC group

To explore the possible mechanism behind *KCNJ14*, we collected the data of correlated genes from Pearson correlation analyses and presented the top five most relevant genes that were positively and negatively correlated with the expression of *KCNJ14* (Additional file 4: Fig. S4a, b). Subsequently, we performed a gene annotation analysis on the genes with expression relationship of *KCNJ14*, and the results showed that there were neutrophil activation, neutrophil-mediated immunity, and Fc receptor-mediated stimulatory signalling pathways in biological processes, including immunoglobulin complex and ficolin-1-rich granules, and molecular functions were immunoglobulin receptor binding, cadherin binding, and cell adhesion molecule binding (Fig. 4a). In addition, KEGG pathway analyses were further applied to obtain more specific information about vital signaling pathways *KCNJ14* participated in, such as the mTOR, NOD-like receptor, and VEGF signalling pathways (Fig. 4b). Finally, we verified the putative signalling pathway in KEGG results and found that knockdown of *KCNJ14* remarkably inhibited the phosphorylation of AKT and mTOR in SW480 (Fig. 4c) and HCT116 (Fig. 4d) cell lines, thus blocking the mTOR signalling pathway, which greatly affected many biological activities of CRC cells, such as proliferation and migration. In addition, we also uploaded the genes with expression relationships with *KCNJ14* to the CMap database to match the inhibitory drug discovery and found that four candidate drugs (thiostrepton, ivermectin, corticosterone, and indoprofen) may have potential value in the treatment of colorectal cancer (Additional file 4: Fig. S4c).

### Relationship between KCNJ14 and immune cell infiltration in colorectal cancer

**Fig. 5:**
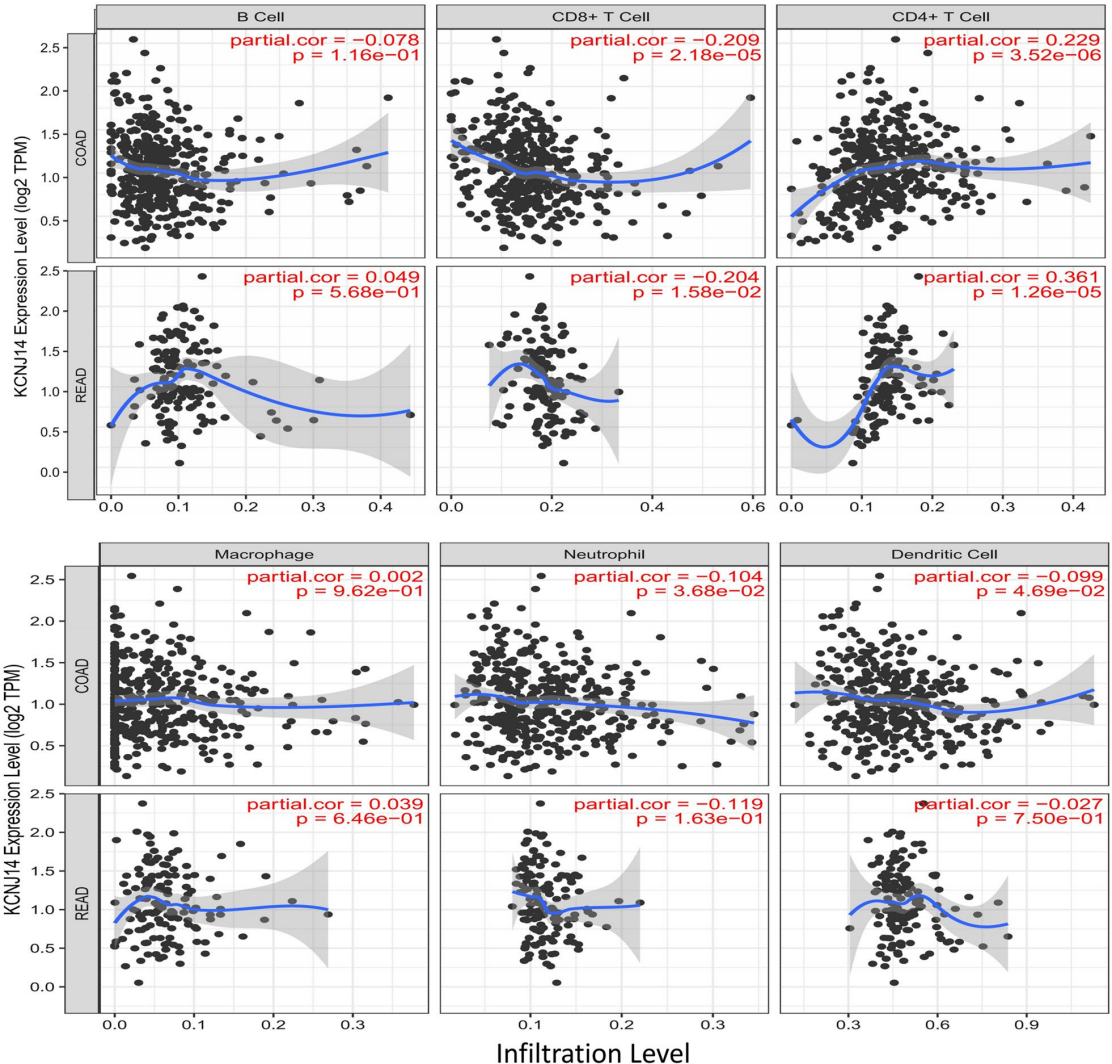
Correlation between *KCNJ14* expression and the infiltration of six immune cells in OAD and READ. The immune cells analysed were B cells, CD8 + T cells, CD4 + T cells, macrophages, neutrophils, and dendritic cells. COAD: Colon adenocarcinoma; READ: Rectum adenocarcinoma

Due to the regulation of pathogenic genes in a variety of ways and the important impact of immune microenvironment on the prognosis of colorectal cancer [18, 19], Therefore, we explored the relationship between *KCNJ14* expression and immune cell infiltration through the TIMER database to reveal the effect of *KCNJ14* expression on immune microenvironment. As shown in Fig. 5, the expression level of *KCNJ14* was positively correlated with CD4 + T cells in both COAD (colon adenocarcinoma) and READ (rectum adenocarcinoma) (*P* = 3.52e-06, COAD; *P* = 1.26e-05, READ) and negatively associated with CD8 + T cells in colorectal cancer (*P* = 2.18e-05, COAD; *P* = 1.58e-02, READ). In addition, *KCNJ14* expression was found to be related to neutrophils and dendritic cells in COAD; however, there were no significant differences in READ, B cells, or macrophages in either COAD or READ. This demonstrates that *KCNJ14* is significantly associated with the tumour immune microenvironment.

## Discussion

Colorectal cancer is responsible for almost 10% of newly diagnosed cancers and related deaths worldwide [20]. The current treatments for CRC have not achieving satisfactory prognosis. Because the mechanisms underlying CRC pathogenesis are not fully understood, targeted therapy is rapidly becoming a key instrument. In this study, we examined the relationship between *KCNJ14* expression and CRC and investigated its potential diagnostic and therapeutic value.

We analysed the TCGA and GEO datasets and conducted a matched-pair analysis. We found that the expression of *KCNJ14* was much higher in colorectal cancer tissues than in adjacent tissues. To verify the reliability of the results, RT-qPCR and western blotting were performed, and results showed that the mRNA and protein levels of *KCNJ14* were significantly higher in colorectal cancer cells than in normal mucosal cells. In addition, the Kaplan–Meier curves showed that high *KCNJ14* expression is correlated with shorter OS in patients with CRC, and multivariate Cox hazard analysis verified that *KCNJ14* is an independent prognostic factor for CRC. Furthermore, we performed a meta-analysis and confirmed that high *KCNJ14* expression is a critical prognostic factor for colorectal cancer. This study is the first to report the prognostic role of high expression of *KCNJ14* in colorectal cancer.

Although the mechanism of action of *KCNJ14* in cancer has not been elucidated, the roles of homologous proteins in various cancers have been extensively reported. For example, *KCNJ2* regulates the expression of *MRP1*/*ABCC1* to regulate cell growth and chemoresistance in small-cell lung cancer [21]. *Kir2.2* can act as a constitutive activator to increase the phosphorylation of RelA, resulting in enhanced NF-kB activity and cell proliferation in cancer [22]. In this study, we demonstrated that high *KCNJ14* expression is positively correlated with tumour stage, suggesting that *KCNJ14* may be a pathogenic gene in colorectal cancer; therefore, we further verified the effect of *KCNJ14* expression on the cellular behaviour of colorectal cancer cells. We knocked down *KCNJ14* in two colorectal cancer cell lines and using in vitro experiments, found that the proliferation and migration ability of cancer cells decreased significantly, thereby verifying the carcinogenic effect of *KCNJ14* in colorectal cancer. Hence, we infer that increased *KCNJ14* expression in colorectal cancer can not only independently reduce the overall survival time of patients, but also enhance the malignant behaviour of colorectal cancer cells.

We next investigated whether increased *KCNJ14* expression in the pathological process of colorectal cancer is regulated by DNA methylation. Previous studies have suggested that aberrant DNA methylation regulates the risk and prognosis of cancer by altering the expression of various genes [23]. Therefore, we detected the methylation level of 11 CpG sites of *KCNJ14* in CRC and applied Pearson correlation analysis to screen out that methylation of the cg17660703 site might increase *KCNJ14* expression. Generally, DNA methylation often leads to the silencing of gene expression; however, with the development of wide-scale analyses of gene expression profiles and DNA methylomes, the positive association between DNA methylation sites and gene expression has been confirmed [24]. The possible mechanisms included negative regulatory element methylation and gene-body DNA methylation [25, 26].

Our results showed that the cg17660703 high methylation status of *KCNJ14* CpG sites is related to unfavourable OS in CRC patients. Similar conclusions have been published to support the theory that aberrant DNA methylation can regulate the activation of oncogenes, leading to malignant progression of cancers [27]. Taken together, our study is the first to demonstrate that the cg17660703 high methylation status of *KCNJ14* CpG sites, along with *KCNJ14* expression, can be a clear indicator of poor prognosis of colorectal cancer patients.

Previous studies have reported that tumour-infiltrating immune cells are recruited to generate a proinflammatory microenvironment, which benefits the progression of CRC and immune cells have become a prognostic marker for colorectal cancer [28, 29]. The TIMER database revealed that *KCNJ14* expression is positively correlated with infiltration of CD4 + T cells and negatively correlatied with that of CD8 + T cells. Consistent with our expectations, CD8 + T cells are considered a preferable prognostic factor for relapse and overall survival in patients with CRC [30]. Increased CD4 + T cell infiltration can enhance the formation of a tumour inhibitory immune microenvironment and lead to a poor prognosis [31]. Our findings indicate that the mRNA level of *KCNJ14* is positively correlated with CD4 + T cells, and that increased expression of *KCNJ14* can lead to poor prognosis in colorectal patients. This suggests that *KCNJ14* and CD4 + T cells may exert a synergistic effect to promote the formation of an inhibitory immune microenvironment in colorectal cancer. Overall, CD4 + T cells play a key role in regulating the cancer immune microenvironment, and our study demonstrated that *KCNJ14* mainly regulates the infiltration of CD4 + and CD8 + cells to influence the development of CRC.

To determine the biological functions of *KCNJ14*, we performed GO annotation analysis. We found that *KCNJ14* is mainly enriched in neutrophil -and immunoglobulin-mediated humoral immunity. Immune responses are believed to participate in the development of colorectal cancer. For example, neutrophil extracellular traps can interact with platelets and endothelial cells to mediate procoagulant activity and contribute to thrombogenesis in colorectal cancer [32]. Moreover, infiltrated neutrophils secrete metalloproteinases to activate latent TGFβ and suppress T-cells, leading to an immunosuppressive microenvironment in colorectal cancer [33]. Finally, immunoglobulin-related mechanisms were also investigated. Immunoglobulin-like receptors on killer cells, such as 3DS1 and 2SD1, contribute to a high risk of CRC [34]. In addition, studies have reported that engineered immunoglobulins with Fc regions can guide activated NK cells against CRC [35]. KEGG analysis also showed that *KCNJ14* participates in cancer-related signalling pathways in colorectal cancer, such as the mTOR, NOD-like receptor, and VEGF signalling pathways. Activation of these pathways can lead to malignant progression of tumour cells [36, 37]; however, we could not verify the influence of *KCNJ14* on these signalling pathways. Therefore, we only performed knockdown *KCNJ14* experiments in two cell lines of colorectal cancer and found that the levels of mTOR signalling pathway-related proteins decreased significantly. This indicates that *KCNJ14* regulates the activity of the mTOR signalling pathway in the pathological process of colorectal cancer.

To validate the possible molecular mechanism of *KCNJ14*, we probed the cancer-related functions of the most positively correlated co-expressed genes. Shadow of prion protein (SPRN) is the one which is accurately reported that the SPRN appears exclusively in leiomyoma in contrast to normal samples, and its overexpression can increase the migratory ability of bladder cancer cells [38]. As a potential splicing factor, SFSWAP can alternatively regulate gene expression, and the cg09170112 methylation site of SFSWAP has been verified to be significantly correlated with colon cancer prognosis [39]. AGAP4 and AGAP6 have not been previously reported; they belong to the GTPase-activating protein family that play a vital role in cancer progression. For example, the homologous protein AGAP1 mainly mediates the migration and invasion of breast cancer cells [40]. Considering its location and function, we speculate that as an ATP-sensitive inward rectifier potassium (K^+^) channel, *KCNJ14* malfunctions in cancer-related microenvironment and consequently activates the following signalling pathways.

Finally, we expanded the treatment options for CRC using CMap analysis and identified four candidate drugs. Thiostrepton was previously considered a thiazole antibiotic and is currently identified as an effective therapeutic drug for colon cancer that targets the oncogenic transcription factor FoxM1 [41]. Ivermectin can reverse chemotherapy resistance in colorectal cancer and breast cancer cells by regulating the EGFR/ERK/Akt/NF-κB pathway [42]. Although the other two agents have not been used for the treatment of CRC, there are indications for their use in enteric diseases and cancers. Corticosterone can inhibit the invasion of bladder cancer cells, and its production in inflamed intestines is increased [43]. Indoprofen can function as a pyruvate kinase M2 (PKM2) inhibitor to facilitate the radiosensitivity of non-small cell lung cancer [44]. Our study expands drug indications and achieves the aim of drug repurposing for a more comprehensive treatment of CRC.

## Conclusions

High expression of *KCNJ14* in CRC can be used as an independent prognostic risk factor, resulting in the poor prognosis in patients with CRC. Therefore, it is expected to become a potential biological target for colorectal treatment. In addition, this study provides novels insights for future studies aiming to investigate the complex pathological process of colorectal cancer and broadens the molecular knowledge on the role of *KCNJ14* in the pathological process of cancer.

## Supplementary Information

**Additional file 1: Fig. S1** The methylation regulation of *KCNJ14* expression. (a) The methylation status of 11 CpG sites of *KCNJ14* in colorectal cancer tissue samples based on the TCGA database. (b) The relationship between cg17660703 methylation and *KCNJ14* expression. (c) The overall survival of CRC patients with high or low methylation status of cg17660703.

**Additional file 2: Fig. S2** Association between *KCNJ14* expression and survival of patients at different stages of CRC. (a) Overall survival of stages I–III CRC patients with high or low expression of *KCNJ14*. (b) Overall survival of stage IV CRC patients with high or low expression of *KCNJ14*.

**Additional file 3: Fig. S3** *KCNJ14* expression in CRC cell lines treated with NC or shKCNJ14. (a) mRNA levels of *KCNJ14* in both HCT116 and SW480 cells treated with NC or shKCNJ14. (b) Protein levels of *KCNJ14* in both HCT116 and SW480 cells treated with NC or shKCNJ14.

**Additional file 4: Fig. S4** Pearson correlation analysis and CMap analysis of *KCNJ14* in CRC. (a)-(b) The 10 most related co-expressed genes of *KCNJ14*, including five positively associated genes (SPRN, AGAP4, BRICD5, SFSWAP and AGAP6) and five negatively associated genes (COPS4, RHOA, CASP3, PAFAH2 and GSKIP). (c)-(f) Pubchem information of four candidate drugs for CRC based on CMap analysis.

## Data Availability

In this study, multiple data were obtained from web-based datasets. The gene expression profiles and clinical information of patients with colorectal cancer in the TCGA database were obtained from the Genomic Data Commons (GDC) Data Portal (https://portal.gdc.cancer.gov/). The GSE50117 and GSE31595 datasets were downloaded from the GEO dataset (http://www.ncbi.nlm.nih.gov/geo/). The correlation between *KCNJ14* expression and immune cell infiltration was obtained from the TIMER database (https://cistrome.shinyapps.io/timer). Finally, candidate therapeutic drugs were screened for co-expressed genes using the CMap database (https://portals.broadinstitute.org/CMap/), and the chemical structure formulas of four drugs, namely corticosterone, indoprofen, ivermectin, and thiostrepton, were searched in the PubChem database (https://pubchem.ncbi.nlm.nih.gov/). All data generated from the analysis of this study are available from the corresponding author upon reasonable request.

## Abbreviations

CRC: Colorectal cancer
TCGA: The Cancer Genome Atlas
GEO: Gene Expression Omnibus
OS: Overall survival
GO: Gene ontology
KEGG: Kyoto Encyclopedia of Genes and Genomes
TIMER: Tumour Immune Estimation Resource
CMap: The connectivity map
NSAIDs: Non-steroidal anti-inflammatory drugs
GDC: Genomic data commons
COAD: Colon adenocarcinoma
READ: Rectum adenocarcinoma
SPRN: Shadow of prion protein
PKM2: Pyruvate kinase M2

## References

[1] Arnold M, Sierra M, Laversanne M, Soerjomataram I, Jemal A, Bray F (2017). Global patterns and trends in colorectal cancer incidence and mortality. Gut.

[2] Jess T, Rungoe C, Peyrin-Biroulet L (2012). Risk of colorectal cancer in patients with ulcerative colitis: a meta-analysis of population-based cohort studies. Clin Gastroenterol Hepatol Off Clin Pract. J Am Gastroenterol Assoc.

[3] Dekker E, Tanis P, Vleugels J, Kasi P, Wallace M (2019). Colorectal cancer. Lancet (Lond, Engl).

[4] Liu Z, Shen F, Wang H, Li A, Wang J, Du L, Liu B, Zhang B, Lian X, Pang B (2020). Abnormally high expression of HOXA2 as an independent factor for poor prognosis in glioma patients. Cell Cycle (Georget, Tex).

[5] Töpert C, Döring F, Derst C, Daut J, Grzeschik K, Karschin A (2000). Cloning, structure and assignment to chromosome 19q13 of the human Kir2.4 inwardly rectifying potassium channel gene (KCNJ14). Mamm Genome Off J Int Mamm Genome Soc.

[6] Tinker A, Aziz Q, Li Y, Specterman M (2018). ATP-sensitive potassium channels and their physiological and pathophysiological roles. Compr Physiol.

[7] Conti M (2004). Targeting K+ channels for cancer therapy. J Exp Ther Oncol.

[8] Sakai H, Shimizu T, Hori K, Ikari A, Asano S, Takeguchi N (2002). Molecular and pharmacological properties of inwardly rectifying K+ channels of human lung cancer cells. Eur J Pharmacol.

[9] Zschüntzsch J, Schütze S, Hülsmann S, Dibaj P, Neusch C (2013). Heterologous expression of a glial Kir channel (KCNJ10) in a neuroblastoma spinal cord (NSC-34) cell line. Physiol Res.

[10] Thuringer D, Chanteloup G, Boucher J, Pernet N, Boudesco C, Jego G, Chatelier A, Bois P, Gobbo J, Cronier L, Solary E, Garrido C (2017). Modulation of the inwardly rectifying potassium channel Kir4.1 by the pro-invasive miR-5096 in glioblastoma cells. Oncotarget.

[11] Pesson M, Volant A, Uguen A, Trillet K, De La Grange P, Aubry M, Daoulas M, Robaszkiewicz M, Le Gac G, Morel A (2014). A gene expression and pre-mRNA splicing signature that marks the adenoma-adenocarcinoma progression in colorectal cancer. PLoS ONE.

[12] Thorsteinsson M, Kirkeby L, Hansen R, Lund L, Sørensen L, Gerds T, Jess P, Olsen J (2012). Gene expression profiles in stages II and III colon cancers: application of a 128-gene signature. Int J Colorectal Dis.

[13] Tan Y, Zhang S, Xiao Q, Wang J, Zhao K, Liu W, Huang K, Tian W, Niu H, Lei T (2020). Prognostic significance of ARL9 and its methylation in low-grade glioma. Genomics.

[14] Lamb J, Crawford E, Peck D, Modell J, Blat I, Wrobel M, Lerner J, Brunet J, Subramanian A, Ross K (2006). The connectivity map: using gene-expression signatures to connect small molecules, genes, and disease. Science.

[15] Huang C, Zhao J, Zhu Z (2021). Prognostic Nomogram of Prognosis-Related Genes and Clinicopathological Characteristics to Predict the 5-Year Survival Rate of Colon Cancer Patients. Front Surg.

[16] Bryan S, Masoud H, Weir H, Woods R, Lockwood G, Smith L, Brierley J, Gospodarowicz M, Badets N (2018). Cancer in Canada: Stage at diagnosis. Health Rep.

[17] Pinho R, Maga E (2021). DNA methylation as a regulator of intestinal gene expression. Br J Nutr.

[18] Yang J, Qi M, Fei X, Wang X, Wang K (2021). LncRNA H19: a novel oncogene in multiple cancers. Int J Biol Sci.

[19] Ganesh K, Stadler Z, Cercek A, Mendelsohn R, Shia J, Segal N, Diaz L (2019). Immunotherapy in colorectal cancer: rationale, challenges and potential. Nat Rev Gastroenterol Hepatol.

[20] Bray F, Ferlay J, Soerjomataram I, Siegel R, Torre L, Jemal A (2018). Global cancer statistics 2018: GLOBOCAN estimates of incidence and mortality worldwide for 36 cancers in 185 countries. CA Cancer J Clin.

[21] Liu H, Huang J, Peng J, Wu X, Zhang Y, Zhu W, Guo L (2015). Upregulation of the inwardly rectifying potassium channel Kir2.1 (KCNJ2) modulates multidrug resistance of small-cell lung cancer under the regulation of miR-7 and the Ras/MAPK pathway. Mol Cancer.

[22] Lee I, Lee S, Kang T, Kang W, Park C (2013). Unconventional role of the inwardly rectifying potassium channel Kir2.2 as a constitutive activator of RelA in cancer. Cancer Res.

[23] Guo Y, Mao X, Qiao Z, Chen B, Jin F (2020). A novel promoter CpG-based signature for long-term survival prediction of breast cancer patients. Front Oncol.

[24] Irizarry R, Ladd-Acosta C, Wen B, Wu Z, Montano C, Onyango P, Cui H, Gabo K, Rongione M, Webster M (2009). The human colon cancer methylome shows similar hypo- and hypermethylation at conserved tissue-specific CpG island shores. Nat Genet.

[25] Dejeux E, Olaso R, Dousset B, Audebourg A, Gut I, Terris B, Tost J (2009). Hypermethylation of the IGF2 differentially methylated region 2 is a specific event in insulinomas leading to loss-of-imprinting and overexpression. Endocr Relat Cancer.

[26] Jjingo D, Conley A, Yi S, Lunyak V, Jordan I (2012). On the presence and role of human gene-body DNA methylation. Oncotarget.

[27] Robertson K (2005). DNA methylation and human disease. Nat Rev Genet.

[28] Kostic A, Chun E, Robertson L, Glickman J, Gallini C, Michaud M, Clancy T, Chung D, Lochhead P, Hold G (2013). Fusobacterium nucleatum potentiates intestinal tumorigenesis and modulates the tumor-immune microenvironment. Cell Host Microbe.

[29] Ye L, Zhang T, Kang Z, Guo G, Sun Y, Lin K, Huang Q, Shi X, Ni Z, Ding N (2019). Tumor-infiltrating immune cells act as a marker for prognosis in colorectal cancer. Front Immunol.

[30] Hu W, Sun R, Chen L, Zheng X, Jiang J (2019). Prognostic significance of resident CD103CD8T cells in human colorectal cancer tissues. Acta Histochem.

[31] Toor S, Murshed K, Al-Dhaheri M, Khawar M, Abu Nada M, Elkord E (2019). Immune checkpoints in circulating and tumor-infiltrating CD4 T cell subsets in colorectal cancer patients. Front Immunol.

[32] Zhang Y, Wang C, Yu M, Zhao X, Du J, Li Y, Jing H, Dong Z, Kou J, Bi Y (2019). Neutrophil extracellular traps induced by activated platelets contribute to procoagulant activity in patients with colorectal cancer. Thromb Res.

[33] Germann M, Zangger N, Sauvain M, Sempoux C, Bowler A, Wirapati P, Kandalaft L, Delorenzi M, Tejpar S, Coukos G (2020). Neutrophils suppress tumor-infiltrating T cells in colon cancer via matrix metalloproteinase-mediated activation of TGFβ. EMBO Mol Med.

[34] Al Omar S, Mansour L, Dar J, Alwasel S, Alkhuriji A, Arafah M, Al Obeed O, Christmas S (2015). The relationship between killer cell immunoglobulin-like receptors and HLA-C polymorphisms in colorectal cancer in a Saudi population. Genet Test Mol Biomarkers.

[35] Schmied B, Riegg F, Zekri L, Grosse-Hovest L, Bühring H, Jung G, Salih H (2019). An Fc-optimized CD133 antibody for induction of natural killer cell reactivity against colorectal cancer. Cancers.

[36] Narayanankutty A (2019). PI3K/ Akt/ mTOR pathway as a therapeutic target for colorectal cancer: a review of preclinical and clinical evidence. Curr Drug Targets.

[37] Liu S, Fan W, Gao X, Huang K, Ding C, Ma G, Yan L, Song S (2019). Estrogen receptor alpha regulates the Wnt/β-catenin signaling pathway in colon cancer by targeting the NOD-like receptors. Cell Signal.

[38] Sahar T, Nigam A, Anjum S, Waziri F, Biswas S, Jain S, Wajid S (2019). Interactome analysis of the differentially expressed proteins in uterine leiomyoma. Anticancer Agents Med Chem.

[39] Yang H, Jin W, Liu H, Wang X, Wu J, Gan D, Cui C, Han Y, Han C, Wang Z (2020). A novel prognostic model based on multi-omics features predicts the prognosis of colon cancer patients. Mol Genet Genomic Med.

[40] Tsutsumi K, Nakamura Y, Kitagawa Y, Suzuki Y, Shibagaki Y, Hattori S, Ohta Y (2020). AGAP1 regulates subcellular localization of FilGAP and control cancer cell invasion. Biochem Biophys Res Commun.

[41] Ju S, Huang C, Huang W, Su Y (2015). Identification of thiostrepton as a novel therapeutic agent that targets human colon cancer stem cells. Cell Death Dis.

[42] Jiang L, Wang P, Sun Y, Wu Y (2019). Ivermectin reverses the drug resistance in cancer cells through EGFR/ERK/Akt/NF-κB pathway. J Exp Clin Cancer Res CR.

[43] Salmenkari H, Issakainen T, Vapaatalo H, Korpela R (2015). Local corticosterone production and angiotensin-I converting enzyme shedding in a mouse model of intestinal inflammation. World J Gastroenterol.

[44] Ye X, Sun Y, Xu Y, Chen Z, Lu S (2016). Integrated in silico-in vitro discovery of lung cancer-related tumor pyruvate kinase M2 (PKM2) inhibitors. Med Chem.

