# Supplementary figures and images for: KCNJ14 knockdown significantly inhibited the proliferation and migration of colorectal cells
Source: BMC Med Genomics. 2022 Sep 13;15:194. doi: 10.1186/s12920-022-01351-4 (PMC9472386; doi:10.1186/s12920-022-01351-4)

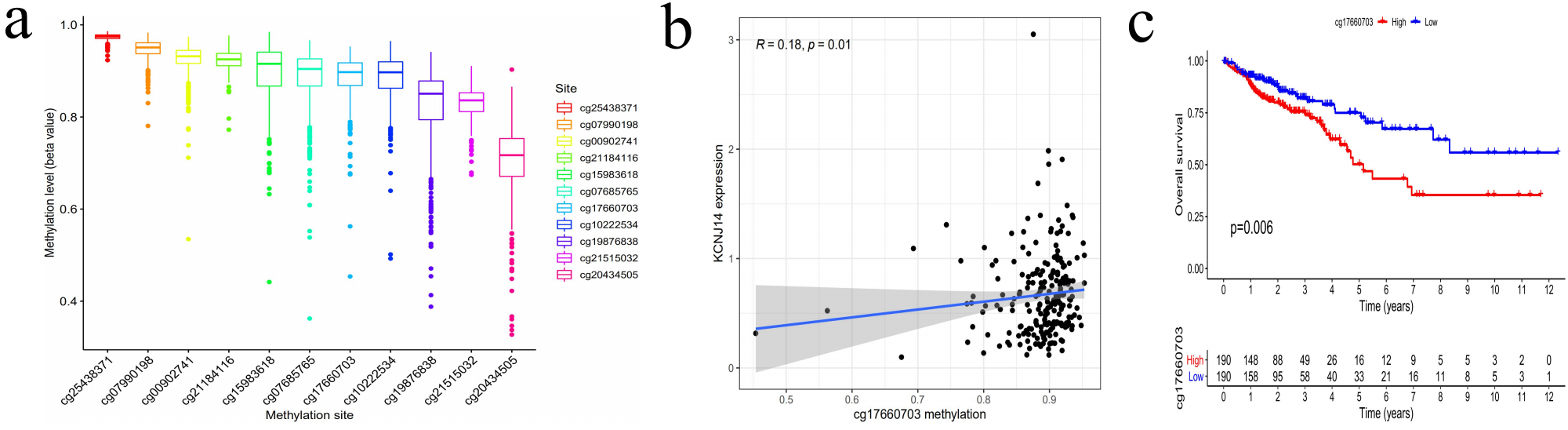

Supplement: Supplementary file 1 — Additional file 1: Fig. S1 The methylation regulation of KCNJ14 expression. (a) The methylation status of 11 CpG sites of KCNJ14 in colorectal cancer tissue samples based on the TCGA database. (b) The relationship between cg17660703 methylation and KCNJ14 expression. (c) The overall survival of CRC patients with high or low methylation status of cg17660703. [file 12920_2022_1351_MOESM1_ESM.tif]

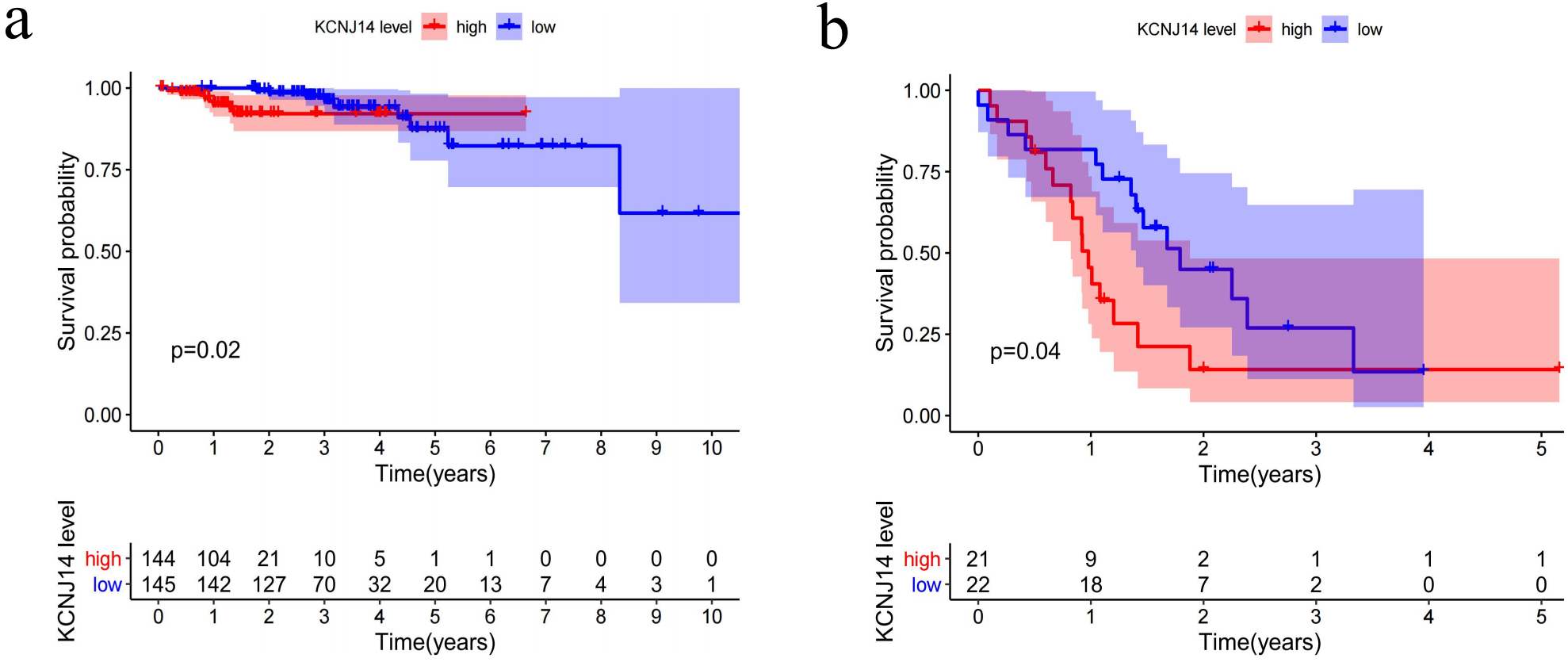

Supplement: Supplementary file 2 — Additional file 2: Fig. S2 Association between KCNJ14 expression and survival of patients at different stages of CRC. (a) Overall survival of stages I–III CRC patients with high or low expression of KCNJ14. (b) Overall survival of stage IV CRC patients with high or low expression of KCNJ14. [file 12920_2022_1351_MOESM2_ESM.tif]

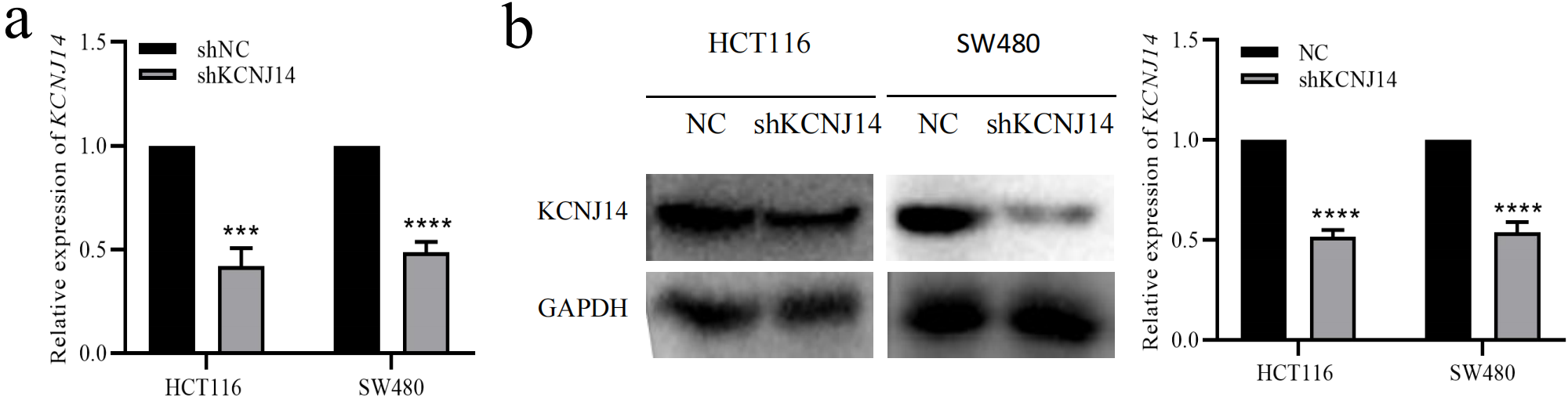

Supplement: Supplementary file 3 — Additional file 3: Fig. S3 KCNJ14 expression in CRC cell lines treated with NC or shKCNJ14. (a) mRNA levels of KCNJ14 in both HCT116 and SW480 cells treated with NC or shKCNJ14. (b) Protein levels of KCNJ14 in both HCT116 and SW480 cells treated with NC or shKCNJ14. [file 12920_2022_1351_MOESM3_ESM.tif]

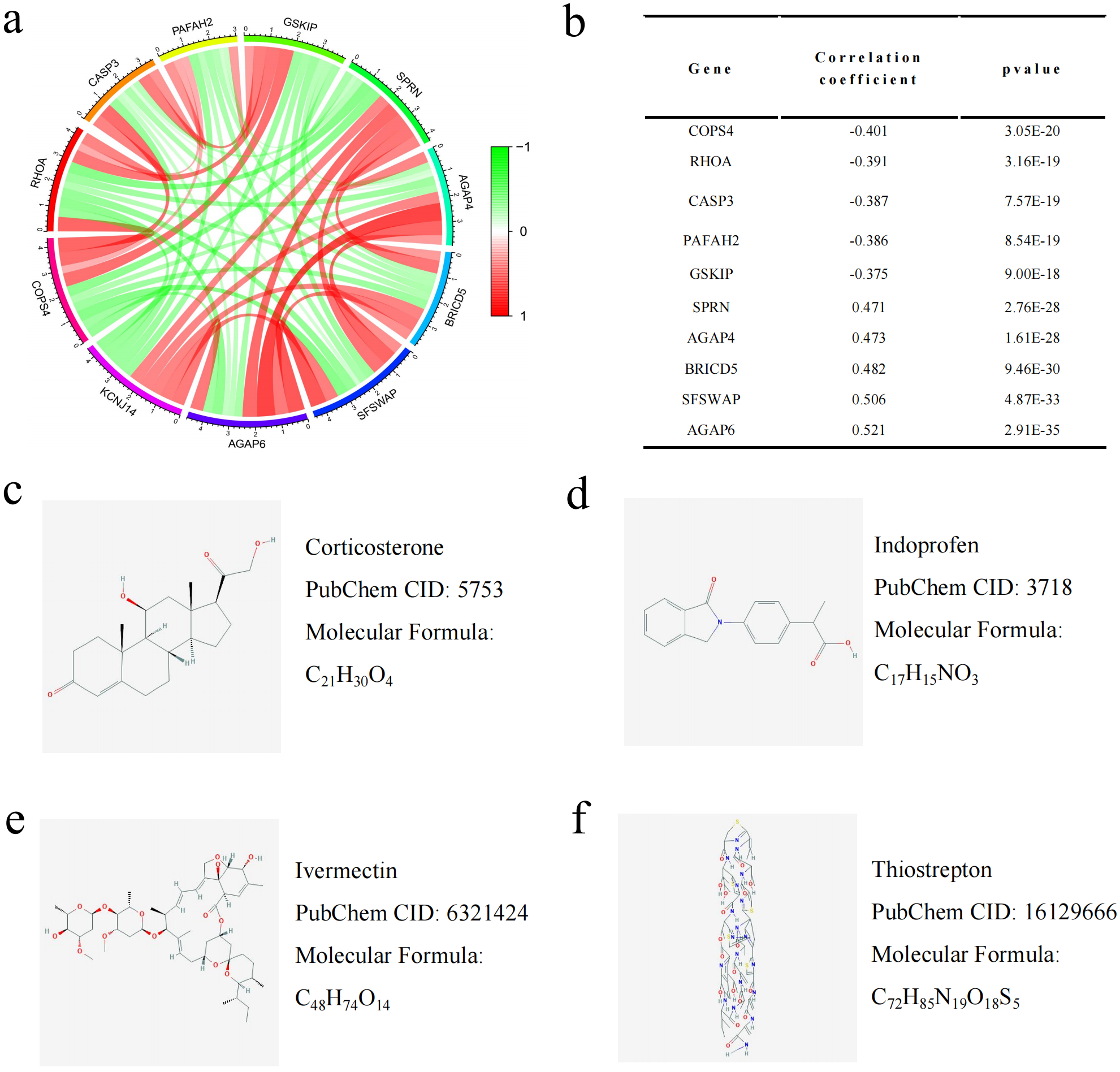

Supplement: Supplementary file 4 — Additional file 4: Fig. S4 Pearson correlation analysis and CMap analysis of KCNJ14 in CRC. (a)-(b) The 10 most related co-expressed genes of KCNJ14, including five positively associated genes (SPRN, AGAP4, BRICD5, SFSWAP and AGAP6) and five negatively associated genes (COPS4, RHOA, CASP3, PAFAH2 and GSKIP). (c)-(f) Pubchem information of four candidate drugs for CRC based on CMap analysis. [file 12920_2022_1351_MOESM4_ESM.tif]
